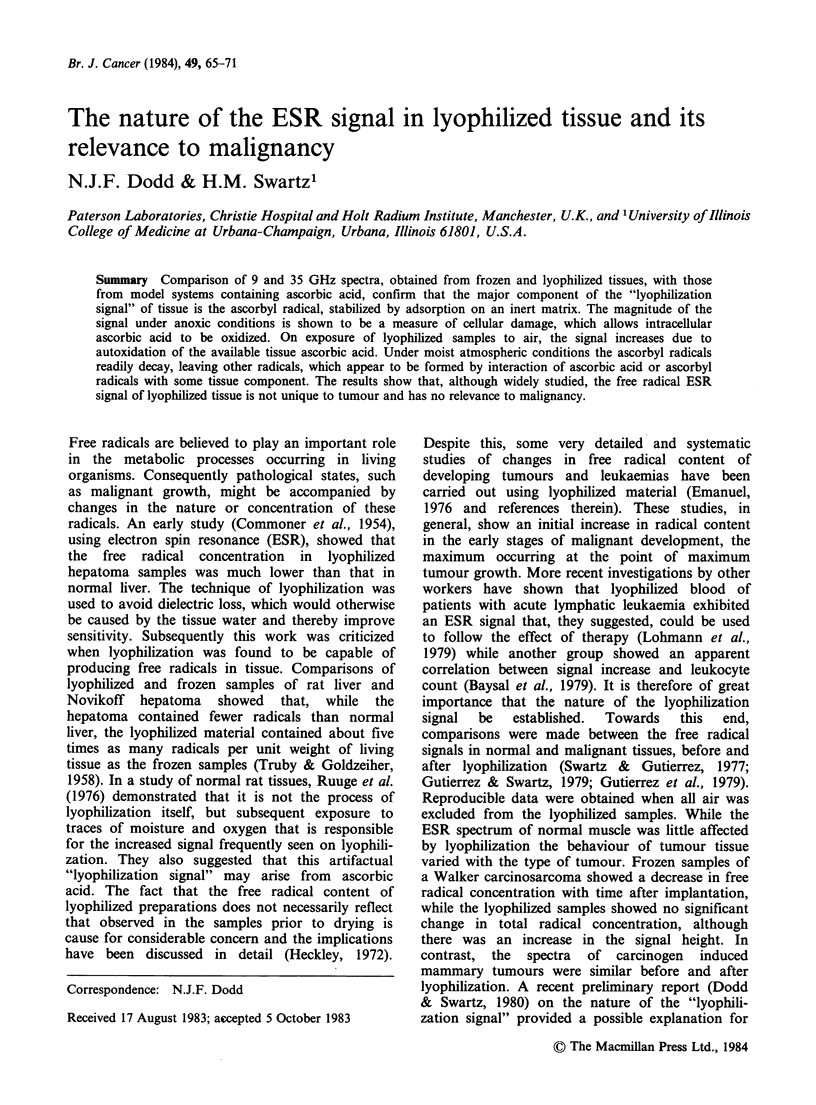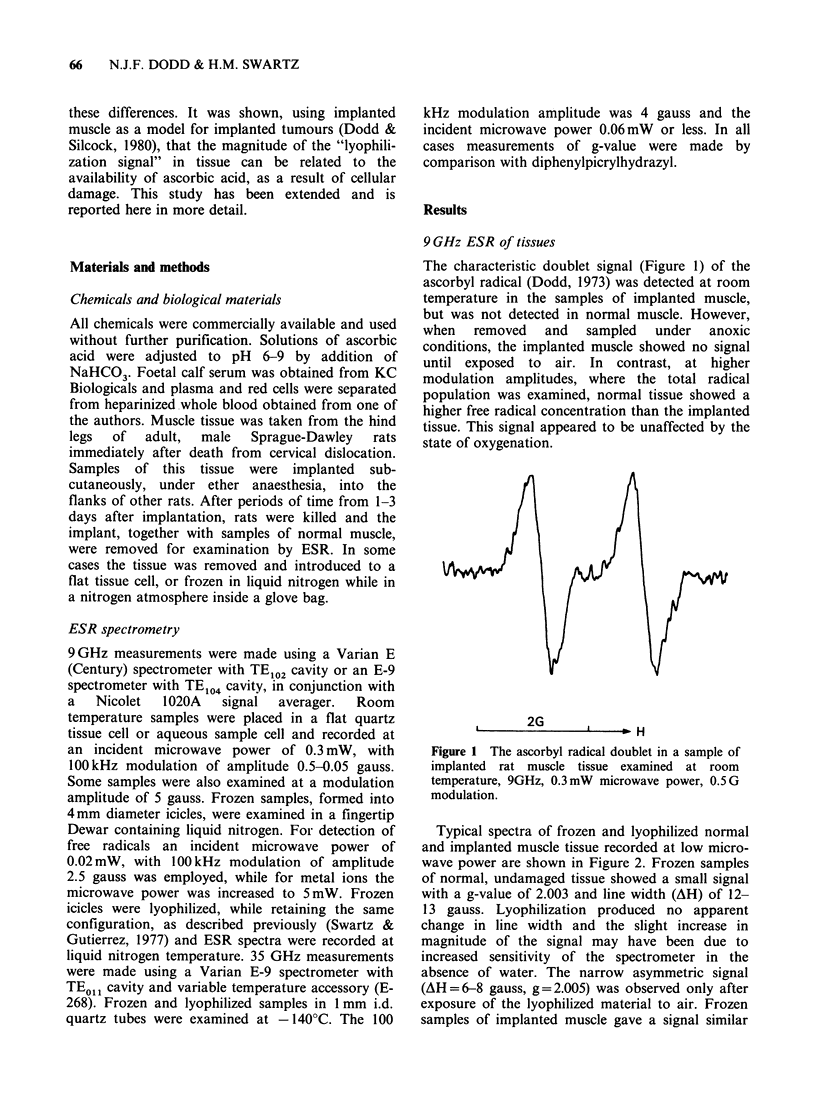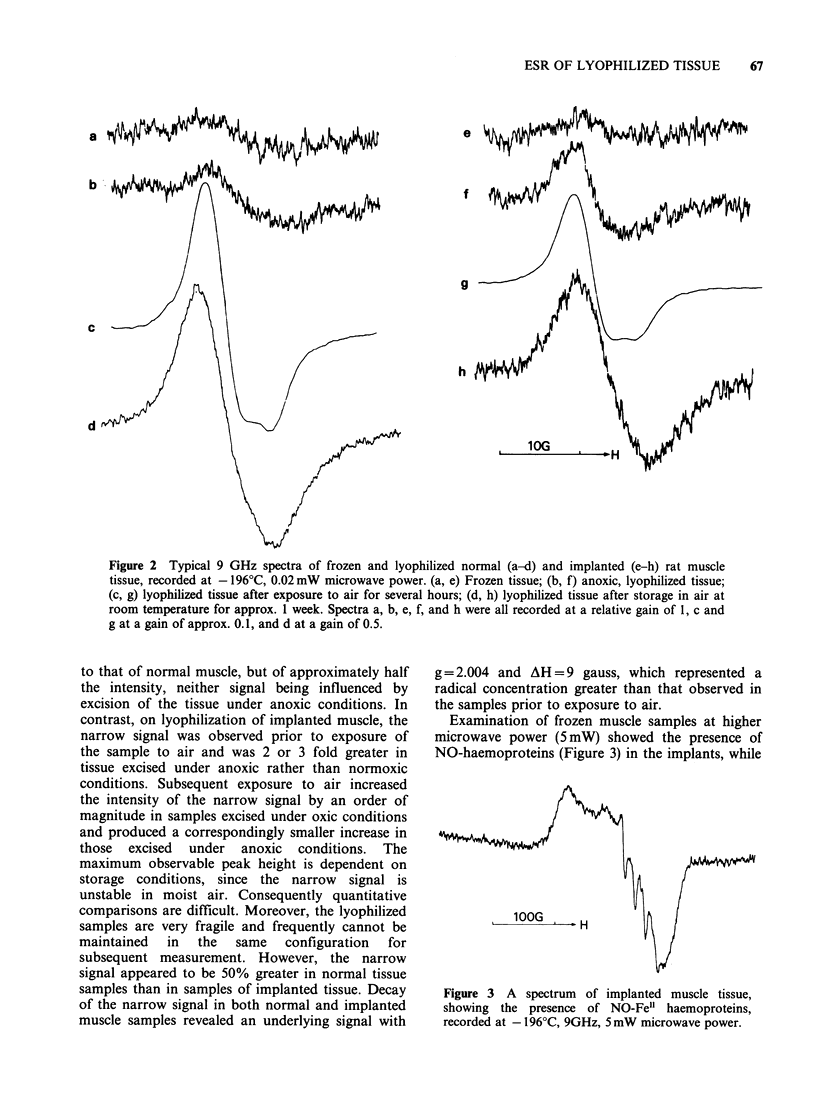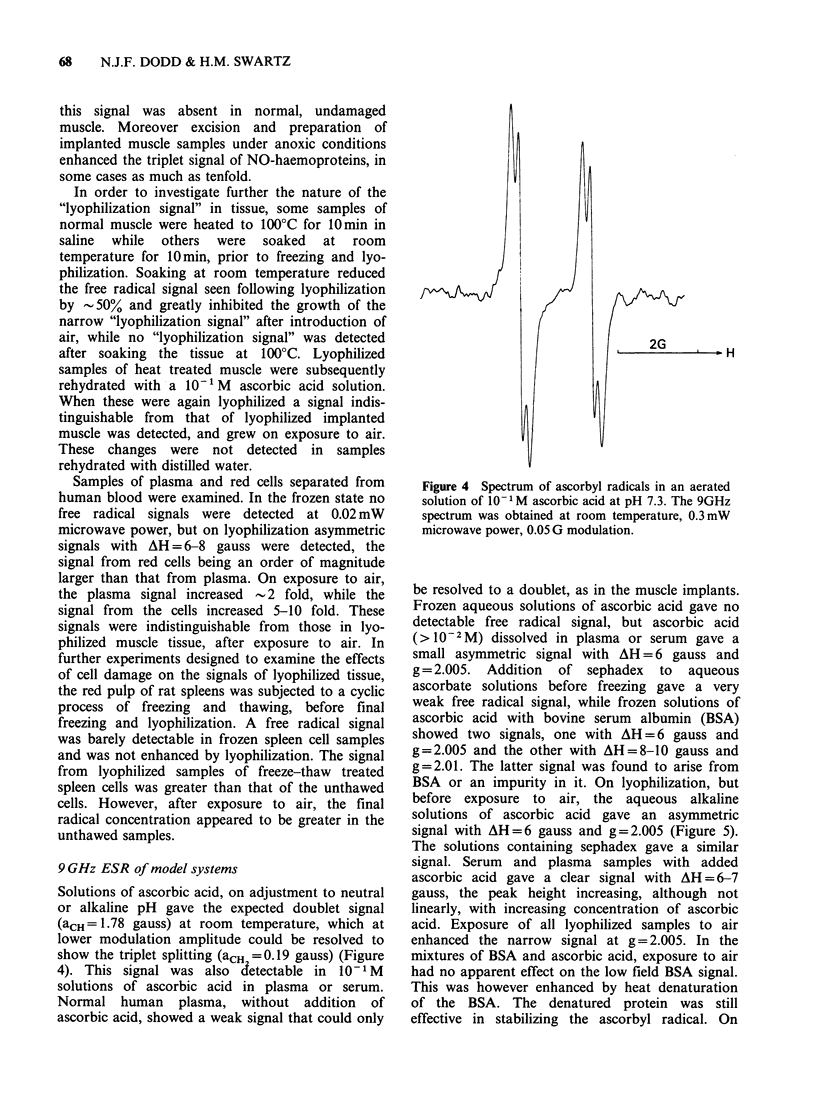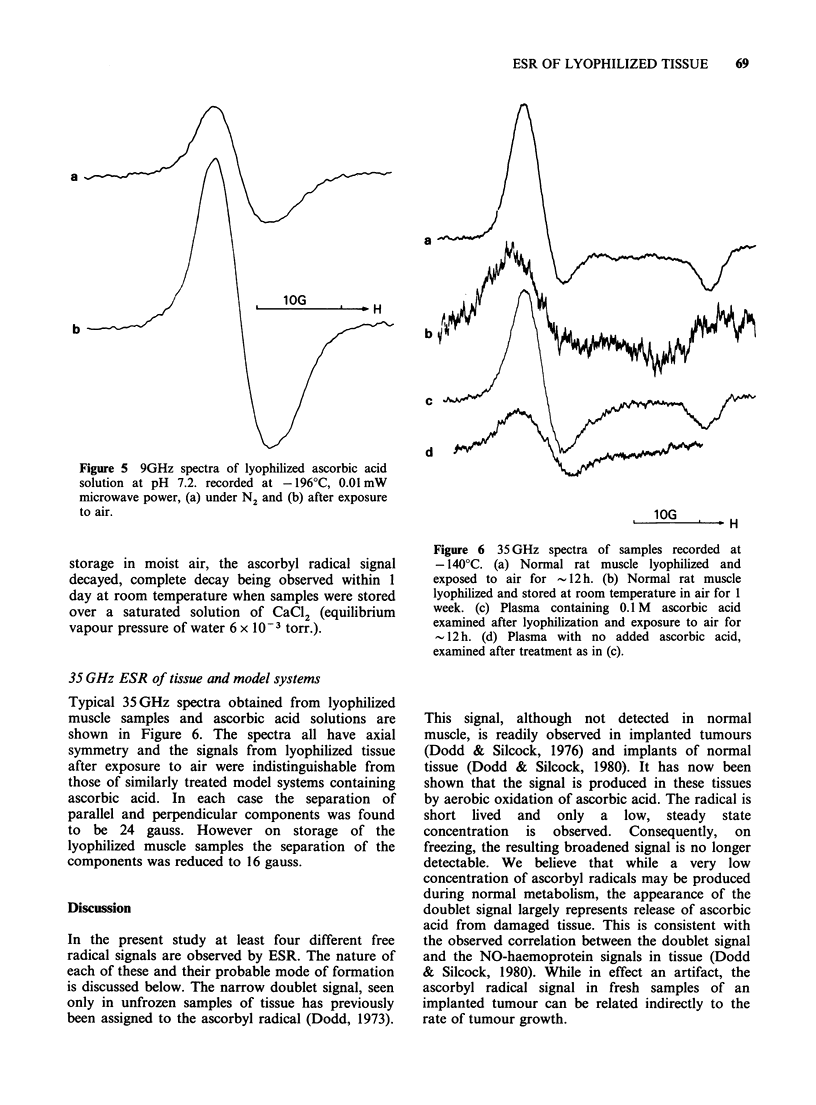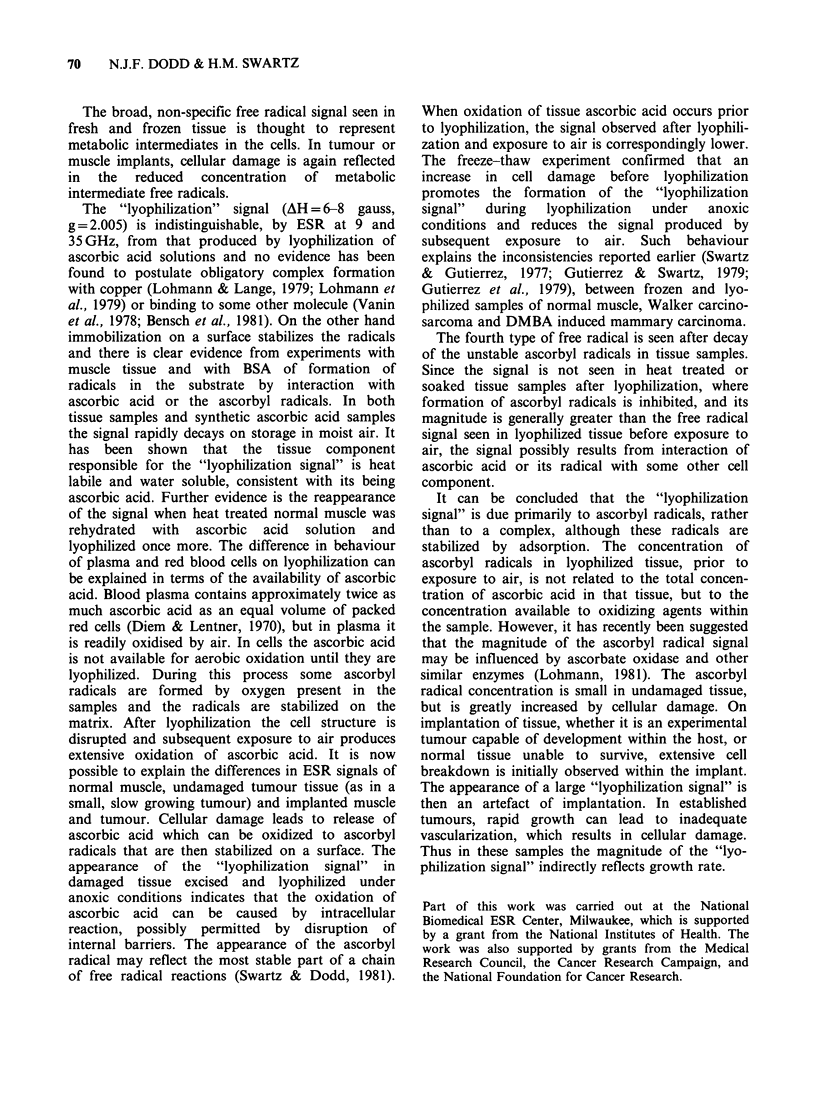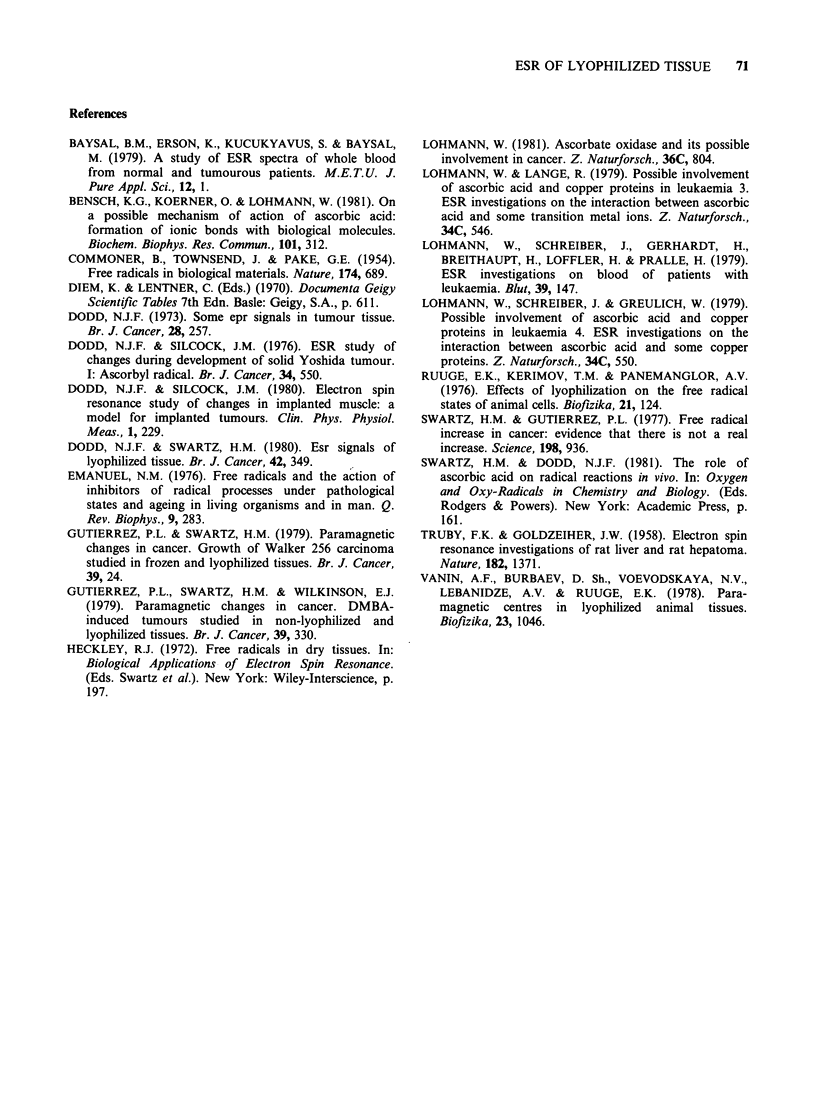# The nature of the ESR signal in lyophilized tissue and its relevance to malignancy.

**DOI:** 10.1038/bjc.1984.10

**Published:** 1984-01

**Authors:** N. J. Dodd, H. M. Swartz

## Abstract

Comparison of 9 and 35 GHz spectra, obtained from frozen and lyophilized tissues, with those from model systems containing ascorbic acid, confirm that the major component of the "lyophilization signal" of tissue is the ascorbyl radical, stabilized by adsorption on an inert matrix. The magnitude of the signal under anoxic conditions is shown to be a measure of cellular damage, which allows intracellular ascorbic acid to be oxidized. On exposure of lyophilized samples to air, the signal increases due to autoxidation of the available tissue ascorbic acid. Under moist atmospheric conditions the ascorbyl radicals readily decay, leaving other radicals, which appear to be formed by interaction of ascorbic acid or ascorbyl radicals with some tissue component. The results show that, although widely studied, the free radical ESR signal of lyophilized tissue is not unique to tumour and has no relevance to malignancy.


*Br. J. Cancer* (1984), **49**, 65–71

### The nature of the ESR signal in lyophilized tissue and its relevance to malignancy

N.J.F. Dodd & H.M. Swartz[1]

*Paterson Laboratories, Christie Hospital and Holt Radium Institute, Manchester, U.K., and [1]University of Illinois College of Medicine at Urbana-Champaign, Urbana, Illinois 61801, U.S.A.*

**Summary** Comparison of 9 and 35 GHz spectra, obtained from frozen and lyophilized tissues, with those from model systems containing ascorbic acid, confirm that the major component of the "lyophilization signal" of tissue is the ascorbyl radical, stabilized by adsorption on an inert matrix. The magnitude of the signal under anoxic conditions is shown to be a measure of cellular damage, which allows intracellular ascorbic acid to be oxidized. On exposure of lyophilized samples to air, the signal increases due to autoxidation of the available tissue ascorbic acid. Under moist atmospheric conditions the ascorbyl radicals readily decay, leaving other radicals, which appear to be formed by interaction of ascorbic acid or ascorbyl radicals with some tissue component. The results show that, although widely studied, the free radical ESR signal of lyophilized tissue is not unique to tumour and has no relevance to malignancy.

Free radicals are believed to play an important role in the metabolic processes occurring in living organisms. Consequently pathological states, such as malignant growth, might be accompanied by changes in the nature or concentration of these radicals. An early study (Commoner *et al.*, 1954), using electron spin resonance (ESR), showed that the free radical concentration in lyophilized hepatoma samples was much lower than that in normal liver. The technique of lyophilization was used to avoid dielectric loss, which would otherwise be caused by the tissue water and thereby improve sensitivity. Subsequently this work was criticized when lyophilization was found to be capable of producing free radicals in tissue. Comparisons of lyophilized and frozen samples of rat liver and Novikoff hepatoma showed that, while the hepatoma contained fewer radicals than normal liver, the lyophilized material contained about five times as many radicals per unit weight of living tissue as the frozen samples (Truby & Goldzeiher, 1958). In a study of normal rat tissues, Ruuge *et al.* (1976) demonstrated that it is not the process of lyophilization itself, but subsequent exposure to traces of moisture and oxygen that is responsible for the increased signal frequently seen on lyophilization. They also suggested that this artifactual "lyophilization signal" may arise from ascorbic acid. The fact that the free radical content of lyophilized preparations does not necessarily reflect that observed in the samples prior to drying is cause for considerable concern and the implications have been discussed in detail (Heckley, 1972).

Correspondence: N.J.F. Dodd

Received 17 August 1983; accepted 5 October 1983

Despite this, some very detailed and systematic studies of changes in free radical content of developing tumours and leukaemias have been carried out using lyophilized material (Emanuel, 1976 and references therein). These studies, in general, show an initial increase in radical content in the early stages of malignant development, the maximum occurring at the point of maximum tumour growth. More recent investigations by other workers have shown that lyophilized blood of patients with acute lymphatic leukaemia exhibited an ESR signal that, they suggested, could be used to follow the effect of therapy (Lohmann *et al.*, 1979) while another group showed an apparent correlation between signal increase and leukocyte count (Baysal *et al.*, 1979). It is therefore of great importance that the nature of the lyophilization signal be established. Towards this end, comparisons were made between the free radical signals in normal and malignant tissues, before and after lyophilization (Swartz & Gutierrez, 1977; Gutierrez & Swartz, 1979; Gutierrez *et al.*, 1979). Reproducible data were obtained when all air was excluded from the lyophilized samples. While the ESR spectrum of normal muscle was little affected by lyophilization the behaviour of tumour tissue varied with the type of tumour. Frozen samples of a Walker carcinosarcoma showed a decrease in free radical concentration with time after implantation, while the lyophilized samples showed no significant change in total radical concentration, although there was an increase in the signal height. In contrast, the spectra of carcinogen induced mammary tumours were similar before and after lyophilization. A recent preliminary report (Dodd & Swartz, 1980) on the nature of the "lyophilization signal" provided a possible explanation for

© The Macmillan Press Ltd., 1984

these differences. It was shown, using implanted muscle as a model for implanted tumours (Dodd & Silcock, 1980), that the magnitude of the "lyophilization signal" in tissue can be related to the availability of ascorbic acid, as a result of cellular damage. This study has been extended and is reported here in more detail.

### Materials and methods

### *Chemicals and biological materials*

All chemicals were commercially available and used without further purification. Solutions of ascorbic acid were adjusted to pH 6–9 by addition of $NaHCO_3$. Foetal calf serum was obtained from KC Biologicals and plasma and red cells were separated from heparinized whole blood obtained from one of the authors. Muscle tissue was taken from the hind legs of adult, male Sprague-Dawley rats immediately after death from cervical dislocation. Samples of this tissue were implanted subcutaneously, under ether anaesthesia, into the flanks of other rats. After periods of time from 1–3 days after implantation, rats were killed and the implant, together with samples of normal muscle, were removed for examination by ESR. In some cases the tissue was removed and introduced to a flat tissue cell, or frozen in liquid nitrogen while in a nitrogen atmosphere inside a glove bag.

### *ESR spectrometry*

9 GHz measurements were made using a Varian E (Century) spectrometer with $TE_{102}$ cavity or an E-9 spectrometer with $TE_{104}$ cavity, in conjunction with a Nicolet 1020A signal averager. Room temperature samples were placed in a flat quartz tissue cell or aqueous sample cell and recorded at an incident microwave power of 0.3 mW, with 100 kHz modulation of amplitude 0.5–0.05 gauss. Some samples were also examined at a modulation amplitude of 5 gauss. Frozen samples, formed into 4 mm diameter icicles, were examined in a fingertip Dewar containing liquid nitrogen. For detection of free radicals an incident microwave power of 0.02 mW, with 100 kHz modulation of amplitude 2.5 gauss was employed, while for metal ions the microwave power was increased to 5 mW. Frozen icicles were lyophilized, while retaining the same configuration, as described previously (Swartz & Gutierrez, 1977) and ESR spectra were recorded at liquid nitrogen temperature. 35 GHz measurements were made using a Varian E-9 spectrometer with $TE_{011}$ cavity and variable temperature accessory (E-268). Frozen and lyophilized samples in 1 mm i.d. quartz tubes were examined at −140°C. The 100 kHz modulation amplitude was 4 gauss and the incident microwave power 0.06 mW or less. In all cases measurements of g-value were made by comparison with diphenylpicrylhydrazyl.

### Results

### *9 GHz ESR of tissues*

The characteristic doublet signal (Figure 1) of the ascorbyl radical (Dodd, 1973) was detected at room temperature in the samples of implanted muscle, but was not detected in normal muscle. However, when removed and sampled under anoxic conditions, the implanted muscle showed no signal until exposed to air. In contrast, at higher modulation amplitudes, where the total radical population was examined, normal tissue showed a higher free radical concentration than the implanted tissue. This signal appeared to be unaffected by the state of oxygenation.

**Figure 1** The ascorbyl radical doublet in a sample of implanted rat muscle tissue examined at room temperature, 9GHz, 0.3 mW microwave power, 0.5 G modulation.

Typical spectra of frozen and lyophilized normal and implanted muscle tissue recorded at low microwave power are shown in Figure 2. Frozen samples of normal, undamaged tissue showed a small signal with a g-value of 2.003 and line width ($\Delta H$) of 12–13 gauss. Lyophilization produced no apparent change in line width and the slight increase in magnitude of the signal may have been due to increased sensitivity of the spectrometer in the absence of water. The narrow asymmetric signal ($\Delta H = 6–8$ gauss, $g = 2.005$) was observed only after exposure of the lyophilized material to air. Frozen samples of implanted muscle gave a signal similar

**Figure 2** Typical 9 GHz spectra of frozen and lyophilized normal (a–d) and implanted (e–h) rat muscle tissue, recorded at $-196°C$, 0.02 mW microwave power. (a, e) Frozen tissue; (b, f) anoxic, lyophilized tissue; (c, g) lyophilized tissue after exposure to air for several hours; (d, h) lyophilized tissue after storage in air at room temperature for approx. 1 week. Spectra a, b, e, f, and h were all recorded at a relative gain of 1, c and g at a gain of approx. 0.1, and d at a gain of 0.5.

to that of normal muscle, but of approximately half the intensity, neither signal being influenced by excision of the tissue under anoxic conditions. In contrast, on lyophilization of implanted muscle, the narrow signal was observed prior to exposure of the sample to air and was 2 or 3 fold greater in tissue excised under anoxic rather than normoxic conditions. Subsequent exposure to air increased the intensity of the narrow signal by an order of magnitude in samples excised under oxic conditions and produced a correspondingly smaller increase in those excised under anoxic conditions. The maximum observable peak height is dependent on storage conditions, since the narrow signal is unstable in moist air. Consequently quantitative comparisons are difficult. Moreover, the lyophilized samples are very fragile and frequently cannot be maintained in the same configuration for subsequent measurement. However, the narrow signal appeared to be 50% greater in normal tissue samples than in samples of implanted tissue. Decay of the narrow signal in both normal and implanted muscle samples revealed an underlying signal with $g=2.004$ and $\Delta H=9$ gauss, which represented a radical concentration greater than that observed in the samples prior to exposure to air.

Examination of frozen muscle samples at higher microwave power (5 mW) showed the presence of NO-haemoproteins (Figure 3) in the implants, while

**Figure 3** A spectrum of implanted muscle tissue, showing the presence of $NO\text{-}Fe^{II}$ haemoproteins, recorded at $-196°C$, 9GHz, 5 mW microwave power.

this signal was absent in normal, undamaged muscle. Moreover excision and preparation of implanted muscle samples under anoxic conditions enhanced the triplet signal of NO-haemoproteins, in some cases as much as tenfold.

In order to investigate further the nature of the "lyophilization signal" in tissue, some samples of normal muscle were heated to 100°C for 10 min in saline while others were soaked at room temperature for 10 min, prior to freezing and lyophilization. Soaking at room temperature reduced the free radical signal seen following lyophilization by ~50% and greatly inhibited the growth of the narrow "lyophilization signal" after introduction of air, while no "lyophilization signal" was detected after soaking the tissue at 100°C. Lyophilized samples of heat treated muscle were subsequently rehydrated with a $10^{-1}$ M ascorbic acid solution. When these were again lyophilized a signal indistinguishable from that of lyophilized implanted muscle was detected, and grew on exposure to air. These changes were not detected in samples rehydrated with distilled water.

Samples of plasma and red cells separated from human blood were examined. In the frozen state no free radical signals were detected at 0.02 mW microwave power, but on lyophilization asymmetric signals with $\Delta H = 6$–$8$ gauss were detected, the signal from red cells being an order of magnitude larger than that from plasma. On exposure to air, the plasma signal increased ~2 fold, while the signal from the cells increased 5–10 fold. These signals were indistinguishable from those in lyophilized muscle tissue, after exposure to air. In further experiments designed to examine the effects of cell damage on the signals of lyophilized tissue, the red pulp of rat spleens was subjected to a cyclic process of freezing and thawing, before final freezing and lyophilization. A free radical signal was barely detectable in frozen spleen cell samples and was not enhanced by lyophilization. The signal from lyophilized samples of freeze–thaw treated spleen cells was greater than that of the unthawed cells. However, after exposure to air, the final radical concentration appeared to be greater in the unthawed samples.

*9 GHz ESR of model systems*

Solutions of ascorbic acid, on adjustment to neutral or alkaline pH gave the expected doublet signal ($a_{CH} = 1.78$ gauss) at room temperature, which at lower modulation amplitude could be resolved to show the triplet splitting ($a_{CH_2} = 0.19$ gauss) (Figure 4). This signal was also detectable in $10^{-1}$ M solutions of ascorbic acid in plasma or serum. Normal human plasma, without addition of ascorbic acid, showed a weak signal that could only be resolved to a doublet, as in the muscle implants. Frozen aqueous solutions of ascorbic acid gave no detectable free radical signal, but ascorbic acid ($> 10^{-2}$ M) dissolved in plasma or serum gave a small asymmetric signal with $\Delta H = 6$ gauss and $g = 2.005$. Addition of sephadex to aqueous ascorbate solutions before freezing gave a very weak free radical signal, while frozen solutions of ascorbic acid with bovine serum albumin (BSA) showed two signals, one with $\Delta H = 6$ gauss and $g = 2.005$ and the other with $\Delta H = 8$–$10$ gauss and $g = 2.01$. The latter signal was found to arise from BSA or an impurity in it. On lyophilization, but before exposure to air, the aqueous alkaline solutions of ascorbic acid gave an asymmetric signal with $\Delta H = 6$ gauss and $g = 2.005$ (Figure 5). The solutions containing sephadex gave a similar signal. Serum and plasma samples with added ascorbic acid gave a clear signal with $\Delta H = 6$–$7$ gauss, the peak height increasing, although not linearly, with increasing concentration of ascorbic acid. Exposure of all lyophilized samples to air enhanced the narrow signal at $g = 2.005$. In the mixtures of BSA and ascorbic acid, exposure to air had no apparent effect on the low field BSA signal. This was however enhanced by heat denaturation of the BSA. The denatured protein was still effective in stabilizing the ascorbyl radical. On

**Figure 4** Spectrum of ascorbyl radicals in an aerated solution of $10^{-1}$ M ascorbic acid at pH 7.3. The 9GHz spectrum was obtained at room temperature, 0.3 mW microwave power, 0.05 G modulation.

**Figure 5** 9GHz spectra of lyophilized ascorbic acid solution at pH 7.2. recorded at −196°C, 0.01 mW microwave power, (a) under $N_2$ and (b) after exposure to air.

**Figure 6** 35 GHz spectra of samples recorded at −140°C. (a) Normal rat muscle lyophilized and exposed to air for ~12 h. (b) Normal rat muscle lyophilized and stored at room temperature in air for 1 week. (c) Plasma containing 0.1 M ascorbic acid examined after lyophilization and exposure to air for ~12 h. (d) Plasma with no added ascorbic acid, examined after treatment as in (c).

storage in moist air, the ascorbyl radical signal decayed, complete decay being observed within 1 day at room temperature when samples were stored over a saturated solution of $CaCl_2$ (equilibrium vapour pressure of water $6 \times 10^{-3}$ torr.).

### *35 GHz ESR of tissue and model systems*

Typical 35 GHz spectra obtained from lyophilized muscle samples and ascorbic acid solutions are shown in Figure 6. The spectra all have axial symmetry and the signals from lyophilized tissue after exposure to air were indistinguishable from those of similarly treated model systems containing ascorbic acid. In each case the separation of parallel and perpendicular components was found to be 24 gauss. However on storage of the lyophilized muscle samples the separation of the components was reduced to 16 gauss.

### Discussion

In the present study at least four different free radical signals are observed by ESR. The nature of each of these and their probable mode of formation is discussed below. The narrow doublet signal, seen only in unfrozen samples of tissue has previously been assigned to the ascorbyl radical (Dodd, 1973). This signal, although not detected in normal muscle, is readily observed in implanted tumours (Dodd & Silcock, 1976) and implants of normal tissue (Dodd & Silcock, 1980). It has now been shown that the signal is produced in these tissues by aerobic oxidation of ascorbic acid. The radical is short lived and only a low, steady state concentration is observed. Consequently, on freezing, the resulting broadened signal is no longer detectable. We believe that while a very low concentration of ascorbyl radicals may be produced during normal metabolism, the appearance of the doublet signal largely represents release of ascorbic acid from damaged tissue. This is consistent with the observed correlation between the doublet signal and the NO-haemoprotein signals in tissue (Dodd & Silcock, 1980). While in effect an artifact, the ascorbyl radical signal in fresh samples of an implanted tumour can be related indirectly to the rate of tumour growth.

The broad, non-specific free radical signal seen in fresh and frozen tissue is thought to represent metabolic intermediates in the cells. In tumour or muscle implants, cellular damage is again reflected in the reduced concentration of metabolic intermediate free radicals.

The "lyophilization" signal ($\Delta H = 6$–$8$ gauss, $g = 2.005$) is indistinguishable, by ESR at 9 and 35 GHz, from that produced by lyophilization of ascorbic acid solutions and no evidence has been found to postulate obligatory complex formation with copper (Lohmann & Lange, 1979; Lohmann *et al.*, 1979) or binding to some other molecule (Vanin *et al.*, 1978; Bensch *et al.*, 1981). On the other hand immobilization on a surface stabilizes the radicals and there is clear evidence from experiments with muscle tissue and with BSA of formation of radicals in the substrate by interaction with ascorbic acid or the ascorbyl radicals. In both tissue samples and synthetic ascorbic acid samples the signal rapidly decays on storage in moist air. It has been shown that the tissue component responsible for the "lyophilization signal" is heat labile and water soluble, consistent with its being ascorbic acid. Further evidence is the reappearance of the signal when heat treated normal muscle was rehydrated with ascorbic acid solution and lyophilized once more. The difference in behaviour of plasma and red blood cells on lyophilization can be explained in terms of the availability of ascorbic acid. Blood plasma contains approximately twice as much ascorbic acid as an equal volume of packed red cells (Diem & Lentner, 1970), but in plasma it is readily oxidised by air. In cells the ascorbic acid is not available for aerobic oxidation until they are lyophilized. During this process some ascorbyl radicals are formed by oxygen present in the samples and the radicals are stabilized on the matrix. After lyophilization the cell structure is disrupted and subsequent exposure to air produces extensive oxidation of ascorbic acid. It is now possible to explain the differences in ESR signals of normal muscle, undamaged tumour tissue (as in a small, slow growing tumour) and implanted muscle and tumour. Cellular damage leads to release of ascorbic acid which can be oxidized to ascorbyl radicals that are then stabilized on a surface. The appearance of the "lyophilization signal" in damaged tissue excised and lyophilized under anoxic conditions indicates that the oxidation of ascorbic acid can be caused by intracellular reaction, possibly permitted by disruption of internal barriers. The appearance of the ascorbyl radical may reflect the most stable part of a chain of free radical reactions (Swartz & Dodd, 1981). When oxidation of tissue ascorbic acid occurs prior to lyophilization, the signal observed after lyophilization and exposure to air is correspondingly lower. The freeze–thaw experiment confirmed that an increase in cell damage before lyophilization promotes the formation of the "lyophilization signal" during lyophilization under anoxic conditions and reduces the signal produced by subsequent exposure to air. Such behaviour explains the inconsistencies reported earlier (Swartz & Gutierrez, 1977; Gutierrez & Swartz, 1979; Gutierrez *et al.*, 1979), between frozen and lyophilized samples of normal muscle, Walker carcinosarcoma and DMBA induced mammary carcinoma.

The fourth type of free radical is seen after decay of the unstable ascorbyl radicals in tissue samples. Since the signal is not seen in heat treated or soaked tissue samples after lyophilization, where formation of ascorbyl radicals is inhibited, and its magnitude is generally greater than the free radical signal seen in lyophilized tissue before exposure to air, the signal possibly results from interaction of ascorbic acid or its radical with some other cell component.

It can be concluded that the "lyophilization signal" is due primarily to ascorbyl radicals, rather than to a complex, although these radicals are stabilized by adsorption. The concentration of ascorbyl radicals in lyophilized tissue, prior to exposure to air, is not related to the total concentration of ascorbic acid in that tissue, but to the concentration available to oxidizing agents within the sample. However, it has recently been suggested that the magnitude of the ascorbyl radical signal may be influenced by ascorbate oxidase and other similar enzymes (Lohmann, 1981). The ascorbyl radical concentration is small in undamaged tissue, but is greatly increased by cellular damage. On implantation of tissue, whether it is an experimental tumour capable of development within the host, or normal tissue unable to survive, extensive cell breakdown is initially observed within the implant. The appearance of a large "lyophilization signal" is then an artefact of implantation. In established tumours, rapid growth can lead to inadequate vascularization, which results in cellular damage. Thus in these samples the magnitude of the "lyophilization signal" indirectly reflects growth rate.

Part of this work was carried out at the National Biomedical ESR Center, Milwaukee, which is supported by a grant from the National Institutes of Health. The work was also supported by grants from the Medical Research Council, the Cancer Research Campaign, and the National Foundation for Cancer Research.

### References

BAYSAL, B.M., ERSON, K., KUCUKYAVUS, S. & BAYSAL, M. (1979). A study of ESR spectra of whole blood from normal and tumourous patients. *M.E.T.U. J. Pure Appl. Sci.*, **12**, 1.

BENSCH, K.G., KOERNER, O. & LOHMANN, W. (1981). On a possible mechanism of action of ascorbic acid: formation of ionic bonds with biological molecules. *Biochem. Biophys. Res. Commun.*, **101**, 312.

COMMONER, B., TOWNSEND, J. & PAKE, G.E. (1954). Free radicals in biological materials. *Nature*, **174**, 689.

DIEM, K. & LENTNER, C. (Eds.) (1970). *Documenta Geigy Scientific Tables* 7th Edn. Basle: Geigy, S.A., p. 611.

DODD, N.J.F. (1973). Some epr signals in tumour tissue. *Br. J. Cancer*, **28**, 257.

DODD, N.J.F. & SILCOCK, J.M. (1976). ESR study of changes during development of solid Yoshida tumour. I: Ascorbyl radical. *Br. J. Cancer*, **34**, 550.

DODD, N.J.F. & SILCOCK, J.M. (1980). Electron spin resonance study of changes in implanted muscle: a model for implanted tumours. *Clin. Phys. Physiol. Meas.*, **1**, 229.

DODD, N.J.F. & SWARTZ, H.M. (1980). Esr signals of lyophilized tissue. *Br. J. Cancer*, **42**, 349.

EMANUEL, N.M. (1976). Free radicals and the action of inhibitors of radical processes under pathological states and ageing in living organisms and in man. *Q. Rev. Biophys.*, **9**, 283.

GUTIERREZ, P.L. & SWARTZ, H.M. (1979). Paramagnetic changes in cancer. Growth of Walker 256 carcinoma studied in frozen and lyophilized tissues. *Br. J. Cancer*, **39**, 24.

GUTIERREZ, P.L., SWARTZ, H.M. & WILKINSON, E.J. (1979). Paramagnetic changes in cancer. DMBA-induced tumours studied in non-lyophilized and lyophilized tissues. *Br. J. Cancer*, **39**, 330.

HECKLEY, R.J. (1972). Free radicals in dry tissues. In: *Biological Applications of Electron Spin Resonance*. (Eds. Swartz *et al.*). New York: Wiley-Interscience, p. 197.

LOHMANN, W. (1981). Ascorbate oxidase and its possible involvement in cancer. *Z. Naturforsch.*, **36C**, 804.

LOHMANN, W. & LANGE, R. (1979). Possible involvement of ascorbic acid and copper proteins in leukaemia 3. ESR investigations on the interaction between ascorbic acid and some transition metal ions. *Z. Naturforsch.*, **34C**, 546.

LOHMANN, W., SCHREIBER, J., GERHARDT, H., BREITHAUPT, H., LOFFLER, H. & PRALLE, H. (1979). ESR investigations on blood of patients with leukaemia. *Blut*, **39**, 147.

LOHMANN, W., SCHREIBER, J. & GREULICH, W. (1979). Possible involvement of ascorbic acid and copper proteins in leukaemia 4. ESR investigations on the interaction between ascorbic acid and some copper proteins. *Z. Naturforsch.*, **34C**, 550.

RUUGE, E.K., KERIMOV, T.M. & PANEMANGLOR, A.V. (1976). Effects of lyophilization on the free radical states of animal cells. *Biofizika*, **21**, 124.

SWARTZ, H.M. & GUTIERREZ, P.L. (1977). Free radical increase in cancer: evidence that there is not a real increase. *Science*, **198**, 936.

SWARTZ, H.M. & DODD, N.J.F. (1981). The role of ascorbic acid on radical reactions *in vivo*. In: *Oxygen and Oxy-Radicals in Chemistry and Biology*. (Eds. Rodgers & Powers). New York: Academic Press, p. 161.

TRUBY, F.K. & GOLDZEIHER, J.W. (1958). Electron spin resonance investigations of rat liver and rat hepatoma. *Nature*, **182**, 1371.

VANIN, A.F., BURBAEV, D. Sh., VOEVODSKAYA, N.V., LEBANIDZE, A.V. & RUUGE, E.K. (1978). Paramagnetic centres in lyophilized animal tissues. *Biofizika*, **23**, 1046.